# Characterization of old RHDV strains by complete genome sequencing identifies a novel genetic group

**DOI:** 10.1038/s41598-017-13902-2

**Published:** 2017-10-19

**Authors:** Ana M. Lopes, Diogo Silvério, Maria J. Magalhães, Helena Areal, Paulo C. Alves, Pedro J. Esteves, Joana Abrantes

**Affiliations:** 1 CIBIO, InBIO - Research Network in Biodiversity and Evolutionary Biology, Universidade do Porto, Campus de Vairão, Rua Padre Armando Quintas, 4485-661 Vairão, Portugal; 2 Departamento de Biologia, Faculdade de Ciências da Universidade do Porto, Rua do Campo Alegre, s/n, 4169-007 Porto, Portugal; 3 Wildlife Biology Program, Department of Ecosystem and Conservation Sciences, University of Montana, Missoula, 59812 Montana USA; 4 Instituto de Investigação e Formação Avançada em Ciências e Tecnologias da Saúde (CESPU), Gandra, Portugal

## Abstract

Rabbit hemorrhagic disease (RHD) is a veterinary disease that affects the European rabbit and has a significant economic and ecological negative impact. In Portugal, rabbit hemorrhagic disease virus (RHDV) was reported in 1989 and still causes enzootic outbreaks. Several recombination events have been detected in RHDV strains, including in the first reported outbreak. Here we describe the occurrence of recombination in RHDV strains recovered from rabbit and Iberian hare samples collected in the mid-1990s in Portugal. Characterization of full genomic sequences revealed the existence of a single recombination breakpoint at the boundary of the non-structural and the structural encoding regions, further supporting the importance of this region as a recombination hotspot in lagoviruses. Phylogenetic analysis showed that in the structural region, the recombinant strains were similar to pathogenic G1 strains, but in the non-structural region they formed a new group that diverged ~13% from known strains. No further reports of such group exist, but this recombination event was also detected in an Iberian hare that was associated with the earliest species jump in RHDV. Our results highlight the importance of the characterization of full genomes to disclose RHDV evolution and show that lagoviruses’ diversity has been significantly undersampled.

## Introduction

In the last decades, the European rabbit (*Oryctolagus cuniculus*) populations have contracted dramatically, particularly in the Iberian Peninsula, the species’ original range, due to the rabbit hemorrhagic disease (RHD)^1^. The disease was first reported in the early 1980s in China^2^, but rapidly disseminated worldwide. RHD causes a severe necrotizing hepatitis and disseminated intravascular coagulation with sudden death of adult rabbits 2–3 days after infection reviewed in^3^. The etiological agent, rabbit hemorrhagic disease virus (RHDV), is a *Lagovirus* of the family *Caliciviridae* ^4^.

The RHDV genomic RNA (gRNA) is ~7.4 kb in length and is organized into two overlapping open reading frames (ORFs)^5^. ORF1 (nucleotides 10–7044) encodes a large polyprotein that is cleaved into several non-structural proteins such as helicase, RNA-dependent RNA polymerase, protease, and the major structural capsid protein VP60^6^. A minor structural protein, VP10, is encoded by ORF2 (nucleotides 7025–7378). The RHDV genome also encodes 5′ and 3′ untranslated regions (5′UTR and 3′UTR, respectively). An additional subgenomic RNA (sgRNA) is present in the RHDV virions and encodes both the major and the minor structural proteins^7^.

The origin of RHDV and its emergence as pathogenic for rabbits remain unclear, with hypotheses including a species jump from a closely related species or the emergence from a pre-existing non-pathogenic lagovirus circulating in leporids^8^. Coincidently, emergence of lagoviruses occurred a few years after the first known introduction attempt in 1966 of a non-native leporid, the Eastern cottontail (*Sylvilagus floridanus*), from the United States of America into Europe^8^. The detection of anti-RHDV antibodies prior to documented outbreaks^9–13^ along with the characterization of a weakly pathogenic and several non-pathogenic strains^14–20^ support the emergence from circulating non-pathogenic lagoviruses. While both hypotheses are not mutually exclusive, they have yet to be confirmed.

RHDV pathogenic strains have been classified into genogroups 1–6 (G1-G6), with G6 representing the first antigenic variant, RHDVa^21,22^. In 2010, a new genetic group of pathogenic strains (RHDV2 or RHDVb) was detected^23^. Phylogenetically, RHDV2 clusters between pathogenic and non-pathogenic strains and diverges more than 15% from the G1-G6 group^8,23^. In addition, it has several features that further place it apart from G1-G6 strains such as a broader host range and the ability to induce RHD in young rabbits (<4 weeks old) in which it ultimately may cause death^24–28^. In the Iberian Peninsula, the first documented RHDV outbreaks date from 1988 and until 2011 they were caused almost exclusively by G1 strains^29–32^. With the arrival of RHDV2, a rapid and complete replacement of Iberian G1 strains was observed that probably resulted from some adaptive advantage of RHDV2 over G1^33,34^.

As other RNA viruses, RHDV capsid gene has a high nucleotide substitution rate^31,35–39^, but less than 10% of genetic divergence is observed between RHDV G1-G6 pathogenic strains. Recombination, along with mutation, is an important mechanism for the evolution of RNA viruses since it uses existing genetic diversity to create new genomic combinations. Recently, a consistent recombination breakpoint located at the boundary between the non-structural genes and the capsid gene was observed for RHDV2 strains from the Iberian Peninsula^40^. This breakpoint was associated with at least two independent recombination events involving non-pathogenic strains and G1 and created different genomic combinations that still persist in the Portuguese wild rabbit populations (Abrantes *et al*., unpublished observations). Other recombination breakpoints, scattered throughout the RHDV genome, had also been identified for classical strains^41–43^.

In this study, we report the occurrence of recombination in RHDV strains recovered from European rabbit and Iberian hare (*Lepus granatensis*) samples collected in the 1990s in Portugal where these two species live in sympatry. This recombination event occurred between the coding region of non-structural proteins of a novel genetic group that diverges ~13% from known pathogenic and non-pathogenic strains and the coding region of structural proteins similar to G1 strains. While no further reports of such novel group exist, the recombination event was associated with the earliest record of RHDV host switch. These results highlight the importance of the characterization of full RHDV genomic sequences to disclose lagoviruses’ diversity and evolution.

## Results

A total of 95 samples were screened for the presence of lagoviruses by amplifying a fragment upstream of the capsid and of the capsid. Samples were randomly chosen and included animals both with and without gross lesions compatible with RHD. PCR-positive samples were sequenced with the amplification primers. Blast searches of the obtained sequences were conducted in public databases to determine their genetic group. For 41 samples, blast searches retrieved similar results for both fragments, and were assigned to G1. In contrast, in four of the sequences, P16, P19, P30 and the previously studied P95^44^, the fragment upstream of the capsid showed the highest identity (~87%) either with G1, G2 or G3-G5 strains, while for the partial sequence of the capsid, the highest identity (~97%) was found with G1 strains (data not shown). Due to this incongruent result, the complete genomic sequences were determined for these four strains.

The obtained sequences were aligned with publicly available full-length genomes (Supplementary Table S1) and the alignment was screened for recombination using RDP (N = 83, 7366 nucleotides). Seven methods (RDP, GENECONV, BootScan, MaxChi, Chimaera, SiScan and 3Seq) detected these four strains as recombinants with strong statistical support (P values < 0.001, Table 1). There was also consistent evidence for a single recombination breakpoint located near the RdRp/VP60 boundary (positions 5242–5399; Table 2) splitting the genome into two distinct subsets, one corresponding to the non-structural coding region and the other to the structural coding region. RDP further identified G1 as the parental genome for the structural subset, but no significant similarity with any of the currently known genetic groups was found for the non-structural subset. This indicated that P16, P19, P30 and P95 are recombinant strains and that the non-structural coding region of the genome of these strains had its origin in strains from a new genetic group.

**Table 1:** RDP results for the putative recombinant strains.

Most closely related parental lineage (genogroup)		Breakpoint (99% confidence interval)	Average *P* value						
NS	S		RDP	GENECONV	BootScan	MaxChi	Chimaera	SiScan	3Seq
Unknown	AST89 (G1)	5342 (5242–5399)	8.63 × 10^−31^	1.16 × 10^−19^	2.78 × 10^−31^	6.21 × 10^−12^	2.85 × 10^−12^	2.87 × 10^−17^	1.42 × 10^−16^

NS: non-structural coding region; S: Structural coding region.

**Table 2:** List of the putative recombinant strains, their year and site of collection, and the gross lesions observed upon necropsy.

Strain	Year/Location	Lesions
P16	1994/Porto (domestic rabbit)	Epistaxis; hemorrhagic trachea; congested lungs, with hemorrhages and edema; slightly congested liver; slightly congested kidney with dark red coloration; abdominal cavity with non-coagulated blood; heart in diastole
P19	1994/Porto (domestic rabbit)	Epistaxis; hemorrhagic trachea; congested lung, with hemorrhages and edema; slightly congested kidney with dark red coloration; slightly congested spleen; heart in diastole with atria filled with blood; liver with a distinct lobular pattern
P30	1994–1995/Porto (domestic rabbit)	Congested lung, with hemorrhages and edema
P95	1996/Torres Novas (Iberian hare^44^)	Hemorrhagic trachea; congested lung, with hemorrhages and edema; congested liver; dilated blood vessels filled with blood; abdominal cavity with blood

**Figure 1:**
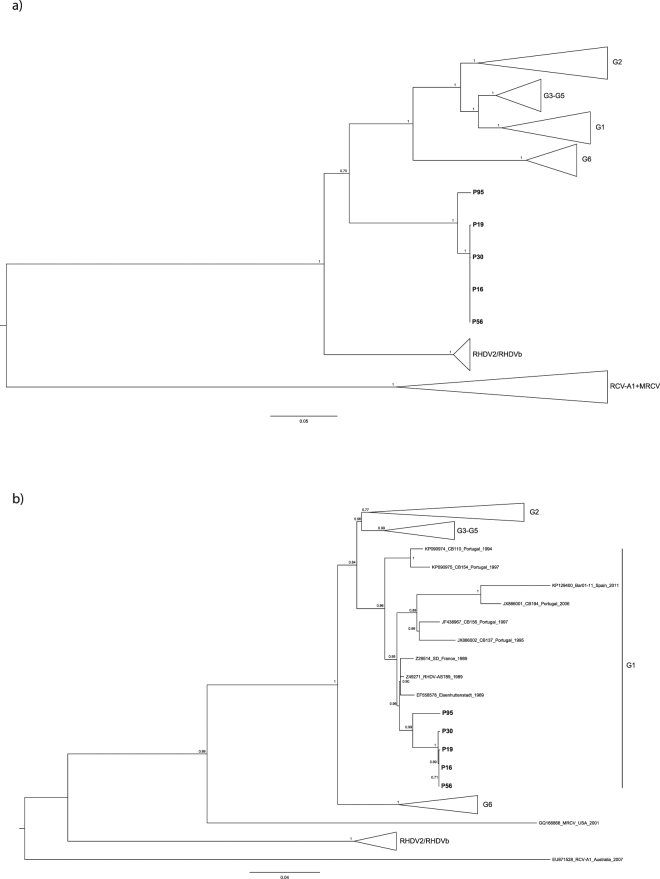
Maximum-likelihood (ML) phylogenetic trees for (**a**) the non-structural fragment (nucleotides 10–5304; nucleotide substitution model GTR + G + I), and (**b**) the structural fragment (VP60 + VP10; nucleotides 5305–7378; nucleotide substitution model K2 + G + I).

To further confirm the recombination event and disclose the evolutionary relationships of the new genetic group, ML phylogenetic trees were constructed for the non-structural (nucleotides 10–5304) and the structural parts (nucleotides 5305–7378). For the non-structural part, the major genetic groups G1, G2, G3-G5, G6 and RHDV2 were identified as highly supported clusters (bootstrap values of 100; Fig. 1a). The four strains sequenced in this work formed a highly supported monophyletic group (bootstrap value of 100) that fell between the G1-G6 and RHDV2 clusters. Thus, they seem to have shared a common ancestor with pathogenic strains rather than with non-pathogenic strains. For the ML tree for the structural part, our strains were closely related with G1 strains from 1989 (bootstrap value of 94; Fig. 1b); interestingly, these strains did not cluster with Portuguese G1 strains from the 1990s, but rather form an exclusive group with older G1 strains circulating in Europe indicating that they are also singular in their structural part. Additionally, MRCV positioning was also different for each genomic part, as recently reported by others^45^.

## Discussion

The distinct positioning of the four strains in the ML phylogenetic trees suggests that they are recombinants and that recombination occurred between G1 (structural part) and a phylogenetically distinct genetic group that had never been reported for the non-structural part. The newly identified recombinant strains diverged more than 13% from currently known pathogenic and non-pathogenic strains (data not shown). While in any recombination event it is difficult to assign which virus is parent or recombinant, particularly when the region involved is completely novel as described here, the most parsimonious explanation is that the strains P16, P19, P30 and P95 recovered in this study are the true recombinants.

In lagoviruses, several recombination events had now been reported^40–43,45^. The Portuguese recombinant strains described in this study present the same modular pattern described for RHDV2 by combining structural and non-structural protein subsets with distinct origins^40^. However, while for RHDV2 the recombination events involved G1 (or non-pathogenic lagoviruses) for the non-structural backbone and RHDV2 in the structural part, for P16, P19, P30 and P95, G1 strains were the donors for the structural backbone (VP60 and VP10). The consistency in the location of the recombination breakpoint observed for these recombination events supports that despite the identification of other breakpoints in the RHDV genome^41,42,45^, the non-structural/structural junction is a recombination hotspot in lagoviruses as observed for other caliciviruses^46–49^.

The biological implication of the different genomic architecture of G1/RHDV2 and the recombinants described in this study for virus fitness are unknown, but G1/RHDV2 recombinants still circulate in Portuguese rabbit populations (Abrantes *et al*., unpublished observations) and were also reported in Azores and Australia^50,51^, suggesting that this recombination event produced viable recombinants. On contrary, it is intriguing that there are no further reports of the strains described here, suggesting the extinction of this lineage. However, these strains were detected in different years and in distant locations. The lack of detection could have been due to a limited sampling, as our screening only included samples from dead rabbits and hares collected between 1993 and 2001. Moreover, this recombination event was detected in a strain recovered from a non-rabbit host in the earliest record of a species jump in RHDV. Indeed, P95 had been previously reported as an Iberian hare found dead in the field which at necropsy presented clinical signs of a *Lagovirus* infection and that was further confirmed to have been due to RHDV^44^. While the role of the recombination in the species jump cannot be assessed, the recombinant strains did cross the species barrier, although they apparently did not sustain further infections.

The evolutionary forces driving recombination in RNA viruses are complex, and recombination has been associated with major changes in virus evolution, emergence and epidemiology. For example, emergence of new pathogenic viruses, expansion of host range and modification of tissue tropism have been observed following recombination events reviewed in^52^. In lagoviruses, recombination seems associated with key events in their evolution. Indeed, the strain recovered from the first reported outbreak in China is the product of a recombination event and RHDV2 emergence was followed by the appearance of several recombinant strains^40,42^. However, as most studies on lagoviruses only focus on the capsid gene, genetic diversity has been significantly underestimated. Hence, our study demonstrates that to fully assess this genus’ evolution, it is crucial to obtain complete genome sequences rather than targeting small regions.

## Methods

### Samples and genome amplification

The samples used in this study were from rabbits and hares found dead in the field or in rabbitries that were collected as part of a surveillance program implemented in Portugal between 1993 and 2001 to monitor RHD. Therefore, no animals were captured, handled or killed in the scope of this study, and thus, most guidelines and legislation for animal experimentation do not apply. Sample collection was conducted in accordance with local legislation and with the permissions and licenses of the National institutions that supervise these activities. The Convention on Biological Diversity and the Convention on the Trade in Endangered Species of Wild Fauna were respected. Animals were submitted to necropsy and samples of liver, lung, heart and spleen were collected and stored at −20 °C. Necropsies were conducted by Dr. Paulo C. Alves. As prospection of carcasses was not performed on a daily basis, it was not possible to accurately determine the time points after death. A portion of the liver was homogenized in a rotor–stator homogenizer (Mixer Mill MM400, Retsch) at 30 Hz for 7 min. Total RNA was extracted with the RNeasy Mini Kit (Qiagen, Hilden, Germany) and reverse transcription was performed using the GRS cDNA synthesis kit (GRISP, Porto, Portugal). Protocols were performed according to the manufacturers’ instructions. Samples were screened by PCR with a forward primer located upstream of the capsid gene and a reverse primer located within the capsid gene^33^. PCR reactions consisted of 0.6 μl of cDNA, 2 pmol of each oligonucleotide, 5 μl Phusion Flash High-Fidelity PCR Master Mix (Thermo Scientific) and water to a final volume of 10 μl. PCR products were purified and sequenced on an automatic sequencer ABI PRISM 310 Genetic Analyzer (PE Applied Biosystems, Foster City, CA, USA) with the forward amplification primer. Blast searches were performed in http://blast.ncbi.nlm.nih.gov/Blast.cgi for genetic group assessment. Four samples with incongruent results in the blast searches, i.e. group assignment for the fragment upstream of the capsid was different from that of the capsid fragment, were then PCR-amplified to obtain full genomic sequences using the genome-walking strategy described in^40,53^. The sequences were deposited in GenBank under the following accession numbers: KJ943791, KY765609, KY765610, KY765611. At necropsy, animals from which the samples were collected presented lesions compatible with RHD (Table 2) such as congested liver, spleen and lungs, hemorrhages in the trachea, lungs and abdominal cavity, and epistaxis. However, gross findings were not confirmed on histopathology.

### Recombination analysis

The obtained full genomic sequences (excluding the UTRs) were aligned using the BioEdit software (version 7.0.9.0)^54^. Full-length RHDV genome sequences available in public databases were retrieved and included in the alignment which produced a final dataset of 84 sequences, with 7369 nucleotides. Sequences previously identified as recombinants were not included^40–42^. The alignment included representatives of all major groups of RHDV: G1-G6, RHDV2, non-pathogenic and weakly pathogenic. The RDP software (version 4.26)^55^ was used to screen the alignment for recombination events under the following parameters: sequences were set to linear, Bonferroni correction and 100 permutations. Only recombination events detected by more than three methods with highest acceptable *P* value 0.05 were considered.

### Phylogenetic analysis

The full-length RHDV genome dataset was partitioned according to the putative recombination breakpoint detected in the recombination analysis as follows: (1) nucleotide positions 10–5304 which included the non-structural proteins and (2) nucleotide positions 5305–7378 which included the major and minor structural proteins. Phylogenetic trees were inferred for both genome partitions using the maximum likelihood (ML) method available in MEGA6^56^. The best model of nucleotide substitution was determined for each partition in MEGA6. The support for each node was determined from 1000 bootstrap replicate ML trees. An additional phylogenetic tree was inferred for VP60 to assign sequences to the major genetic groups. This dataset included all publicly available non-recombinant VP60 sequences and was composed of 271 sequences of 1740 nucleotides. This phylogenetic tree was estimated using the same phylogenetic approach as described above.

## Electronic supplementary material

Supplementary Table S1
