# Supplementary material for: Characterization of old RHDV strains by complete genome sequencing identifies a novel genetic group
Source: Sci Rep. 2017 Oct 19;7:13599. doi: 10.1038/s41598-017-13902-2 (PMC5648873; doi:10.1038/s41598-017-13902-2)
Supplement: Supplementary file 1 — Supplementary Table S1 [file 41598_2017_13902_MOESM1_ESM.pdf]

Characterization of old RHDV strains by complete genome sequencing identifies a novel genetic group

Ana M. Lopes, Diogo Silvério, Maria J. Magalhães, Helena Areal, Paulo C. Alves, Pedro J. Esteves, Joana Abrantes

Supplementary Table S1. GenBank accession numbers of the sequences used for the analyses.

Group	GenBank accession number_strain identification
G1	KP090974_CB110_Portugal_1994
	KP090975_CB154_Portugal_1997
	JF438967_CB156_Portugal_1997
	EF558578_Eisenhuttenstadt_1989
	JX886001_CB194_Portugal_2006
	JX886002_CB137_Portugal_Alpiarça_1995
	Z29514_SD_France_1989
	Z49271_RHDV-AST89_1989
	KP129400_Bar01-11_Spain_2011
G2	DQ189078_Saudi_Arabia
	EU003579_Italy90_1990
	EU003580_Korea90_1990
	KP144789_Pd_Poland/1989
	KP144790_KGM_Poland/1988
	KT006721_NZL/Canterbury/Lincoln/517/2013
	KT006722_NZL/Canterbury/MackenzieBasin/95/2012
	KT006723_NZL/HawkesBay/BoundaryStream/94/2012
	KT006724_NZL/Canterbury/Hawarden/320/2013
	KT006725_NZL/Otago/Queensberry/74/2013
	KT006726_NZL/Otago/Taras/61/2014
	KT006727_NZL/Otago/Luggate/67/2014
	KT006728_NZL/Otago/Alexandra/64/2014
	KT006729_NZL/Otago/Wanaka/63/2014
	KT006730_NZL/Canterbury/MackenzieBasin/322/2013
	KT006731_AUS/NSW/Hall/WAT1-13/2007
	KT006732_AUS/NSW/Murrumbateman/BlueGums/2014
	KT006733_AUS/WA/Gnowangerup/1999
	KT006734_AUS/SA/FlindersRanges/1999
	KT006735_AUS/WA/Bunbury/2000
	KT006736_AUS/SA/Robe/2000
	KT006737_AUS/SA/FlindersRanges/2002
	KT006738_AUS/SA/OneTreeHill/2003

	KT006739_AUS/SA/Coorong/2004
	KT006740_AUS/SA/BillaKallina/2004
	KT006741_AUS/SA/Woodcroft/2005
	KT006742_AUS/SA/FlindersRanges/2006
	KT006743_AUS/TAS/SouthArm/2007
	KT006744_AUS/SA/Hillbank/2008
	KT006745_AUS/SA/Bulgania/2009
	KT006746_AUS/WA/WonganHills/2012
	KT006747_AUS/NSW/Grafton/2013
	KT280058_BlueGums-2
	KT280059_BLMT-2
	KT344770_AUS/CSIRO/Master
	KT344771_AUS/CSIRO/Working
	KT344772_AUS/CSIRO/Release
	KT344773_AUS/EMAI/Batch-1A
	KT344774_AUS/EMAI/Batch-1D
G3-G5	DQ189077_Bahrain_2001
	EF363035_clonepJG-RHDV-DD06_1999
	EF558575_Ascot_UK_1992
	EF558576_Jena_Germany
	EF558577_Meiningen_Germany_1993
	KP144792_BLA_Poland/1994
	X87607_BS89_1989
G6	AB300693_Hokkaido/2002/JPN_2002
	AF258618_Iowa2000_2000
	AY523410_CD/China
	DQ205345_JX/CHA/97_1997
	DQ280493_ChinaWHNRH
	EF558581_Erfurt_2000
	EF558582_Dachswald_2000
	EF558583_Triptis_1996
	EF558584_Rossi_2002
	EU003578_IN-05_2005
	EU003581_NY-1_2001
	EU003582_UT-01_2001
	HM623309_NJ-2009_China_2009
	JF412629_HYD_China_Heilongjiang_2005
	KF677011_STR2012_Poland/2012
MRCV	GQ166866_MRCV_USA_2001
RCV-A1	EU871528_RCV-A1_Australia_2007
RHDV2	KM878681_RHDV-N11_Spain
	KM979445_CBVal16_Portugal_2012
	KP129395_Rij06-12_Spain
	KP129396_Seg08-12_Spain
	KP129397_Tar06-12_Spain

	KP129399_Zar06-12_Spain
	KP129398_Zar11-11_Spain_2010
